# Parapyruvate Induces Neurodegeneration in C57BL/6JNarl Mice via Inhibition of the α-Ketoglutarate Dehydrogenase Complex

**DOI:** 10.1021/acsomega.3c09469

**Published:** 2024-01-25

**Authors:** Inn Lee, Tuzz-Ying Song, Chien-Lin Chen, Jiann-Jou Yang, Nae-Cherng Yang

**Affiliations:** † Department of Nutrition, Chung Shan Medical University, No. 110, Sec. 1, Jianguo N. Rd., Taichung 40201, Taiwan; ‡ Department of Medicinal Botanicals and Foods on Health Applications, Da-Yeh University, No. 168, University Rd., Dacun, Changhua 51591, Taiwan; § Department of Biomedical Sciences, Chung Shan Medical University, No. 110, Sec. 1, Jianguo N. Rd., Taichung 40201, Taiwan; ∥ Department of Nutrition, Chung Shan Medical University Hospital, No. 110, Sec. 1, Jianguo N. Rd., Taichung 40201, Taiwan

## Abstract

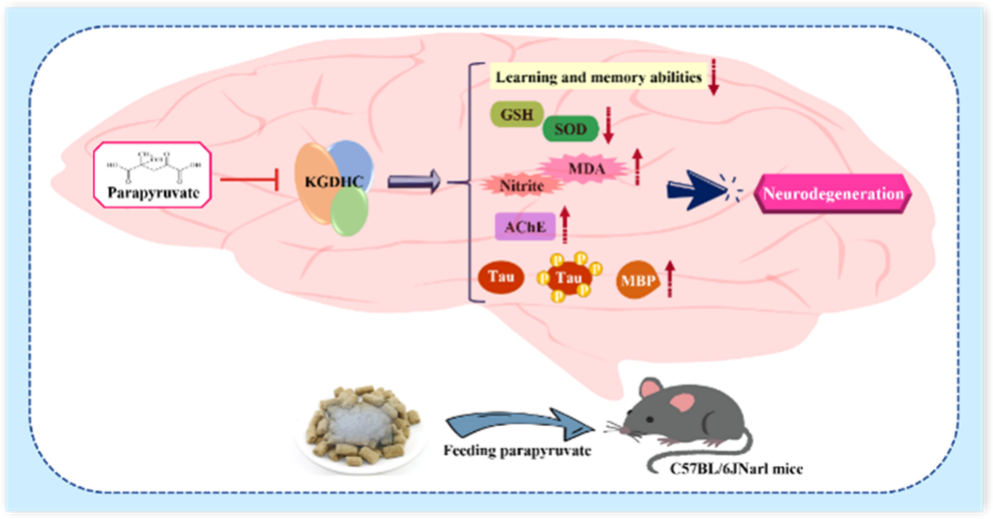

Parapyruvate is a substance commonly found in commercial dietary supplements of calcium pyruvate (DSCP) that inhibits the α-ketoglutarate dehydrogenase complex (KGDHC) and has been shown to induce senescence in human Hs68 cells. However, it is unknown whether parapyruvate can induce neurodegeneration. In this study, the parapyruvate content in DSCP was converted to an equivalent dose for mice and administered to the C57BL/6JNarl mice at doses around the equivalent dose for 69 days, including 5, 50, and 500 mg/kg/day. The Morris water maze (MWM) task and the active avoidance test were conducted to assess the learning and memory ability in mice, and then brain tissues were collected for biochemical analyses. The results demonstrated that parapyruvate significantly impaired the learning and memory ability, decreased the KGDHC activity, and promoted the oxidative stress and acetylcholinesterase (AChE) activity in mice in a dose-dependent manner. Additionally, parapyruvate induced Tau and phosphorylated Tau (p-Tau) aggregation at dosages ≥5 mg/kg/day and increased the myelin basic protein (MBP) expression at a dosage of 500 mg/kg/day. These results suggest that the equivalent dose of parapyruvate can induce neurodegeneration in the C57BL/6JNarl mice.

## 1 Introduction

Parapyruvate is formed by the aldol addition reaction of two molecules of pyruvate^1^ and is commonly found in commercial dietary supplements of calcium pyruvate (DSCP).^2−4^ Parapyruvate is a reversibly competitive inhibitor of the α-ketoglutarate dehydrogenase complex (KGDHC) with a structure similar to α-ketoglutarate (α-KG), which is the substrate of KGDHC.^5^ Parapyruvate can also be used as an inhibitor of the tricarboxylic acid cycle (TCA).^6^ Previous studies have shown that the decreased KGDHC activity is significantly correlated with many neurodegenerative diseases,^7,8^ such as Alzheimer’s disease (AD)^9−13^ and Parkinson’s disease (PD).^14−17^ In addition, the KGDHC impairment has been proposed as one of the mechanisms causing neurodegenerative disorders.^18−25^ In our previous study, we found that the parapyruvate content in the five brands of DSCP ranged from 1.4 ± 0.1 to 10.6 ± 0.2%.^2^ Furthermore, we also found that 0.5 mM parapyruvate can induce senescence in human fibroblastic Hs68 cells by inhibiting the KGDHC activity.^2^ However, it is still unknown whether the parapyruvate content in DSCP induces neuronal injury in animals.

It has been proposed that the activity of KGDHC is decreased due to the increased oxidative stress during aging, leading to neurodegenerative diseases.^26,27^ This is because KGDHC is more sensitive to oxidative damage than other proteins in mitochondria.^8,28^ The decreased activity of KGDHC impacts the energy metabolism and causes abnormalities in the mitochondrial function, further leading to neurodegeneration.^28^ The impaired mitochondrial function results in the reduced cellular ATP production and increased the reactive oxygen species (ROS) generation.^29^ It is also known that the oxidative stress is one of the main mechanisms that induce neurodegenerative diseases.^30^ Therefore, we are interested in investigating whether the daily intake dosage of parapyruvate from DSCP is sufficient to inhibit the KGDHC activity, inducing oxidative stress and further causing neurodegeneration in mice.

Spatial learning and memory impairment are common behavioral indicators used to assess the neurodegeneration status in mice.^31−33^ In our previous study, we performed the Morris water maze (MWM) test and active avoidance test to assess the effect of ergothioneine on the spatial learning and memory function in the C57BL/6JNarl mice.^34^ However, the effect of parapyruvate on inducing behavioral changes in the MWM and active avoidance tests is unknown. Additionally, the excessive ROS activates the microglia and leads to neuroinflammation, further causing the deposition of amyloid β peptide (Aβ)^35−37^ and the aggregation of the Tau protein.^38^ Thus, the deposition of Aβ and the aggregation of Tau protein are the most significant and typical hallmarks of AD.^39^ However, whether parapyruvate can induce the deposition of Aβ and the aggregation of Tau protein in mice is unknown.

In this study, we are interested in investigating whether the estimated daily intake dosage of parapyruvate from DSCP can induce neuronal injury in the C57BL/6JNarl mice. Therefore, the estimated daily intake dosage of parapyruvate in humans, deduced from the parapyruvate content in DSCP, was converted to an equivalent dosage for mice. We hypothesized that the equivalent dosage of parapyruvate could inhibit the activity of the KGDHC, induce oxidative stress, and induce neurodegeneration in the C57BL/6JNarl mice. The equivalent dosage of parapyruvate was estimated as 50 mg/kg/day. Thus, the low, medium, and high dosages of parapyruvate administration were set as 5, 50, and 500 mg/kg/day and orally administrated to the mice. The MWM and active avoidance tests were conducted to evaluate the loss of spatial learning and memory ability after the administration of parapyruvate. The activities of KGDHC and superoxide dismutase (SOD) and the levels of reduced glutathione (GSH), malondialdehyde (MDA), and nitrite were detected. In addition, the deposition of Aβ and the aggregation of the Tau protein in the brain tissue were detected by immunohistochemical (IHC) staining to detect the expression of Aβ42, Tau, and phosphorylated Tau (p-Tau) in the cortex and hippocampus areas. Additionally, d-galactose (DG) was used as the positive control because it could induce oxidative damage to promote brain aging and induce the cognitive dysfunction in mice.^40,41^ Since it generally takes 8 to 10 weeks for DG to induce brain aging, the administration time of parapyruvate was set as 69 days. In addition, the alternative neurodegenerative indicators, including the acetylcholinesterase (AChE) activity^42^ and the expression of myelin basic protein (MBP) in the brain tissues,^39,43^ were also evaluated. Moreover, there have been no reported findings in the literature to date regarding the ability of parapyruvate to cross the blood–brain barrier (BBB). However, despite differences in the carbon number, the literature suggests that other dicarboxylic acids like glutaric acids, 3-hydroxyglutaric acid, and methylmalonic acid possess the capability to penetrate the BBB.^44^ Therefore, we assume here that parapyruvate can cross the BBB. If this study were to observe any significant neurotoxicity associated with parapyruvate, it could serve as indirect evidence supporting its potential to cross the BBB.

## 2 Results

### 2.1 Effects of Parapyruvate and DG on the Body Weight in C57BL/6Narl Mice

**Figure 1:**
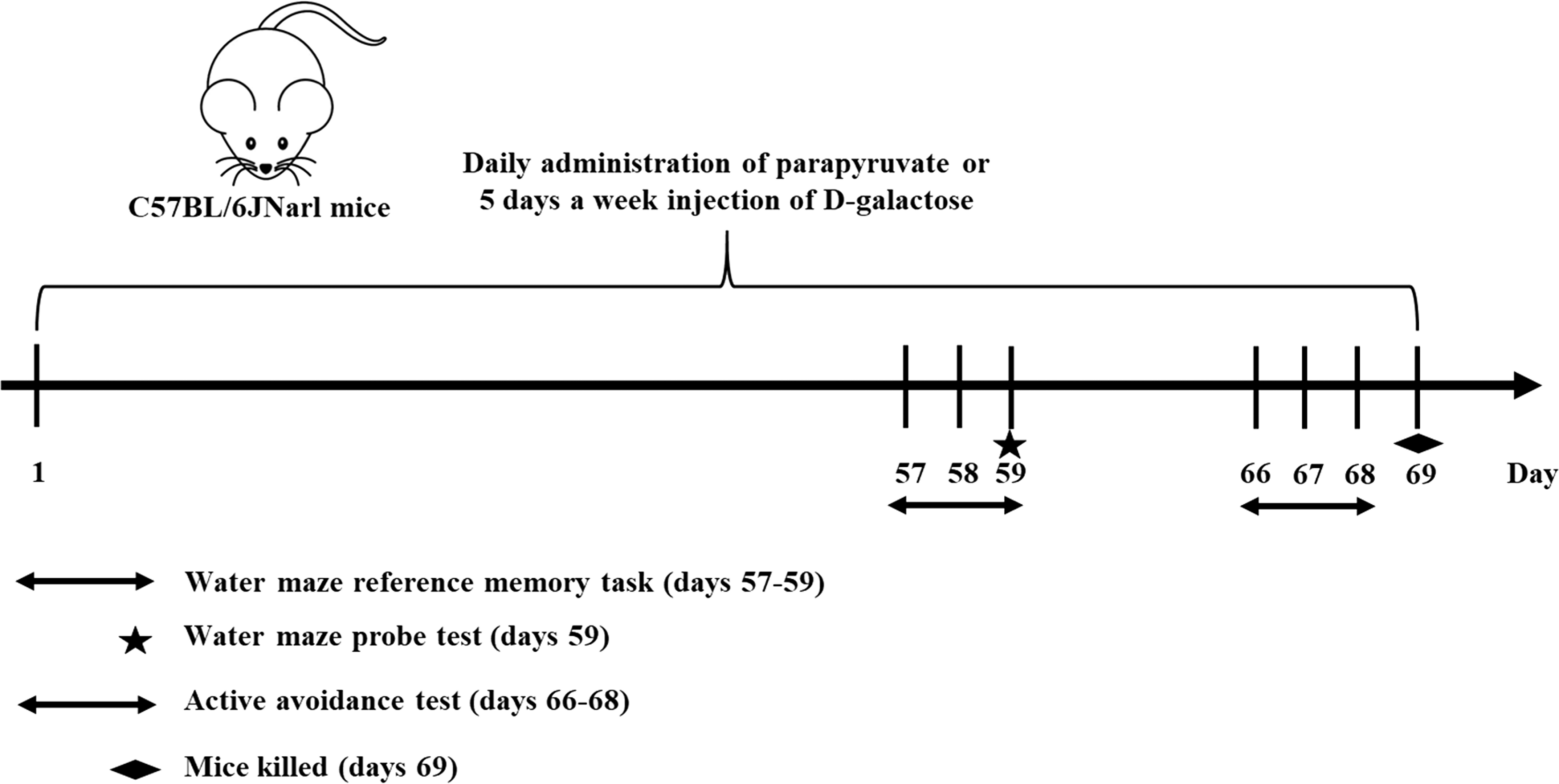
Experimental scheme for the treatment of parapyruvate or d-galactose (DG) as well as the Morris water maze tests and the active avoidance test.

**Figure 2:**
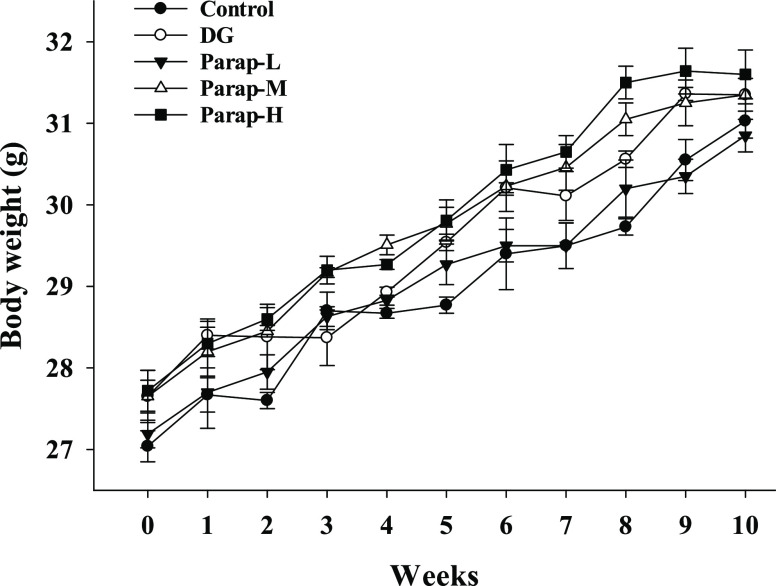
Effects of parapyruvate and DG on body weight in the C57BL/6JNarl mice. Parapyruvate was administered in the daily diet at dosages of 5 (the low dose of parapyruvate; Parap-L), 50 (the medium dose of parapyruvate; Parap-M), and 500 mg/kg body weight/day (the high dose of parapyruvate; Parap-H). DG was dissolved in 0.9% normal saline and injected subcutaneously at a dosage of 120 mg/kg body weight/day 5 days/week. Data are expressed as means ± SD (*n* = 10 mice/group).

The experimental design is shown in Figure 1. The changes in body weight during the administration for all of the groups are shown in Figure 2. Because the group effect on the changes in body weight could be affected by the difference of body weight between the groups at the baseline (i.e., day zero), the percentage change from baseline was used to reveal the effects of parapyruvate and DG on the changes in body weight. The percentage change from the baseline in body weight during the administration for all of the groups is shown in Figure S1. The results of the two-way repeated measures ANOVA indicated that both the main effects of time and group were significant (*p* < 0.001). According to the Tukey test, no significant difference was observed between Parap-M and Parap-H, while both groups were significantly different from others. DG had no significant difference with both the control and Parap-L groups but was significantly different from other groups. The interaction between the group and time was also statistically significant (*p* < 0.05). These results indicated that parapyruvate at medium to high dosages, but not DG, had a significant effect on the weight gain over time compared to the control.

### 2.2 Morris Water Maze Task

#### 2.2.1 Reference Memory Test

**Figure 3:**
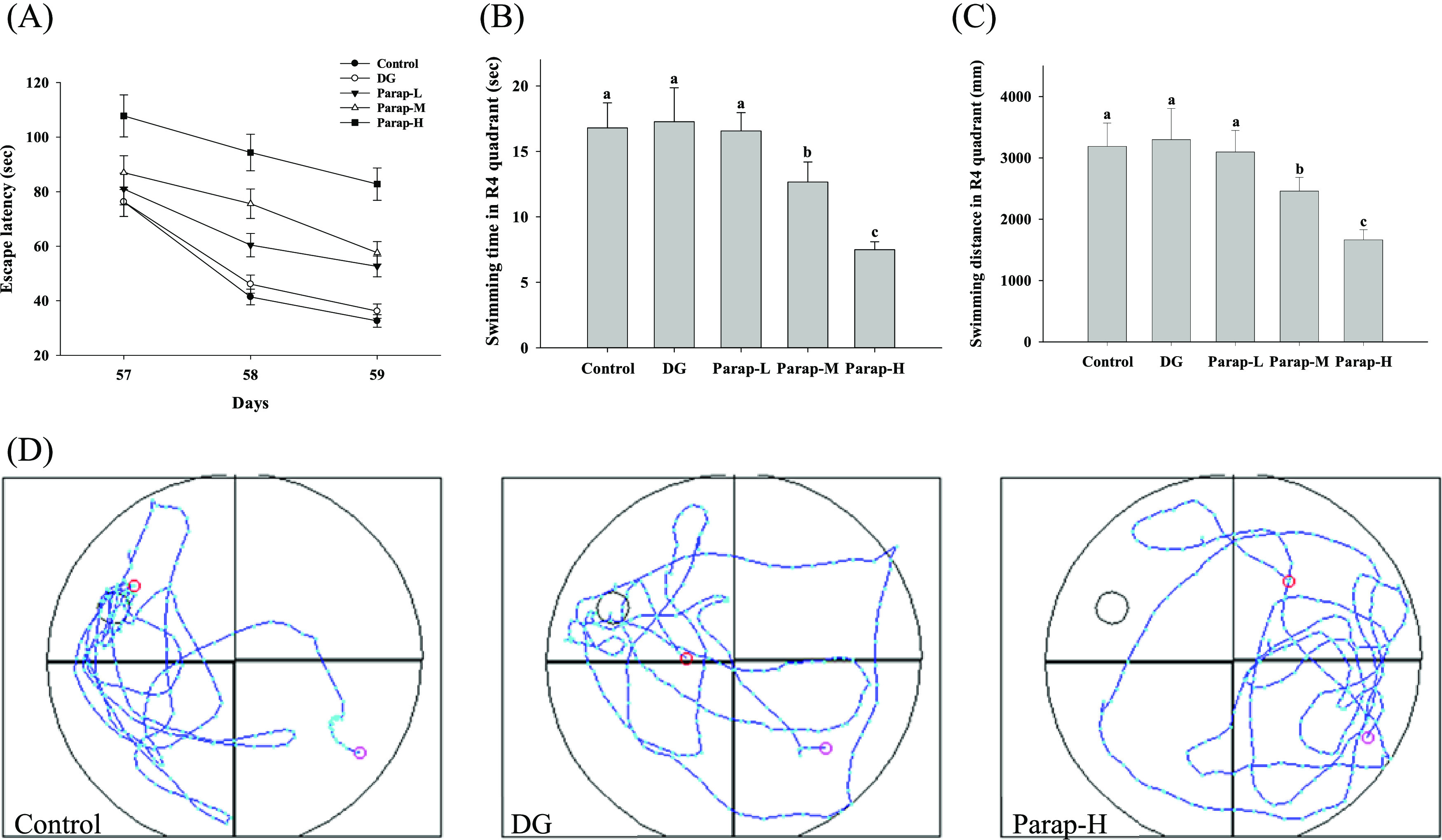
Effects of parapyruvate and DG on the learning and memory ability in the Morris water maze reference memory test and probe test in the C57BL/6Narl mice. After being administered with low (Parap-L), medium (Parap-M), and high (Parap-H) dosages of parapyruvate and 120 mg/kg body weight/day of DG, (A) the reference memory tests were conducted on days 57–59 and the escape latency is as shown. For the probe tests, (B) the total time and (C) the total distance spent on staying in the fourth quadrant, where the escape platform was placed in, measured within 60 s are as shown. (D) Swimming routes of the mouse are as shown. Data are expressed as means ± SD (*n* = 10 mice per group). For (A), data are analyzed by the two-way repeated measures ANOVA followed by the Tukey test. For (B, C), data not sharing an alphabetic letter are significantly different (*p* < 0.05).

The learning ability of mice was assessed using a reference memory test in the Morris water maze task. The results of the escape latency in the Morris water maze test are as presented in Figure 3A. The two-way repeated measures ANOVA results indicated that the main effects of time and group, as well as the interaction of both, were all statistically significant (*p* < 0.001). According to the Tukey test, all pairwise differences were significant for all of the groups except for the Control versus DG (*p* = 0.803). The results indicated that the escape latency gradually decreased with time for each group. Additionally, the parapyruvate-administered groups exhibited a dose-dependent increase in the escape latency on days 57–59, while DG had no effect on the escape latency (Figure 3A). These results suggest that the effect of the administered dosages of parapyruvate impaired the learning ability of the mice in a dose-dependent manner. The swimming speed in the MWM test of mice was from 187 to 210 mm/s, and there was no difference between all of the groups (Table S1).

#### 2.2.2 Probe Test

The probe test in the Morris water maze task was used to evaluate the effects of parapyruvate on memory impairment in the mice. The results showed that the mice administered medium and high doses of parapyruvate significantly took less time to swim in the quadrant where the platform existed (Figure 3B) and swam shorter distances (Figure 3C). However, mice supplemented with a low dosage of parapyruvate and DG swam in the platform-existing quadrant with no significant difference in time and distance as compared to the control (Figure 3B,C). In addition, the corresponding swimming path maps in the probe test are also shown in Figure 3D. The results indicated that a dosage of parapyruvate above 50 mg/kg/day could reduce the retention memory of the mice.

### 2.3 Active Avoidance Test

**Figure 4:**
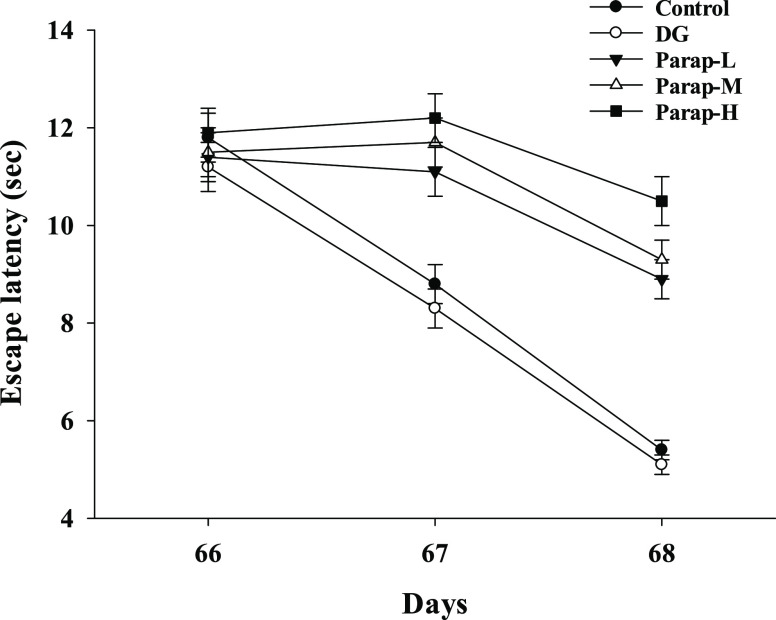
Effects of parapyruvate and DG on the active avoidance test in the C57BL/6Narl mice. After being administered with low (Parap-L), medium (Parap-M), and high (Parap-H) dosages of parapyruvate and 120 mg/kg body weight/day of DG, the mice were subjected to an active avoidance electric shock test for three consecutive days on days 66–68, and the time spent is as shown. Each mouse was tested 5 times a day for three consecutive days in each group. Data are expressed as means ± SD (*n* = 10 mice per group) and are analyzed by two-way repeated measures ANOVA followed by the Tukey test.

The escape latency was used to evaluate the learning and memory ability in the active avoidance test (Figure 4). Results from the two-way repeated measures ANOVA indicated that the main effects of time and group, as well as the interaction of both, were all statistically significant (*p* < 0.001). According to the Tukey test, all pairwise differences were significant for all of the groups except the Control versus DG (*p* = 0.218) and Parap-L versus Parap-M (*p* = 0.425). The results showed that all of the groups of mice in the active avoidance tests exhibited a progressive decrease in the escape latency, representing the time taken by the mice to escape to the other chamber before the electronic shock. However, the Parap-H group exhibited a less progressive decrease in the escape latency as compared to other groups. The escape latencies were significantly increased in the parapyruvate-administered groups (including the low, medium, and high doses) but not in the DG group when compared to the control group on days 66–68. These results suggested that parapyruvate impairs the learning and memory ability in the C57BL/6JNarl mice, but DG does not.

### 2.4 Effects of Parapyruvate on the KGDHC Activity in the Brain

**Figure 5:**
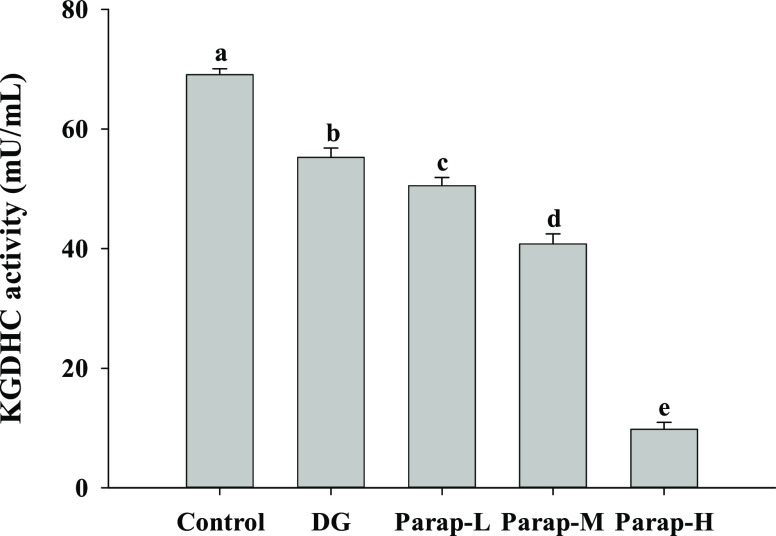
Effects of parapyruvate and DG on the KGDHC activity in brain tissues of the C57BL/6Narl mice. After being administered with low (Parap-L), medium (Parap-M), and high (Parap-H) dosages of parapyruvate and 120 mg/kg body weight/day of DG, the KGDHC activity in the brain of randomly selected mice (*n* = 5 mice per group) was measured and is as shown. Values (means ± SD) not sharing an alphabetic letter are significantly different (*p* < 0.05).

The results showed that the KGDHC activity in the brain was significantly decreased by different dosages of parapyruvate compared to the control in a dose-dependent manner (Figure 5). Oral administration of the low, medium, and high dosages of parapyruvate reduced the KGDHC activity in the brain by 26.9, 40.9, and 85.8%, respectively, compared to the control. The subcutaneous injection of DG reduced the KGDHC activity by 19.9%.

### 2.5 Effects of Parapyruvate on the Antioxidant Status in the Brain

**Figure 6:**
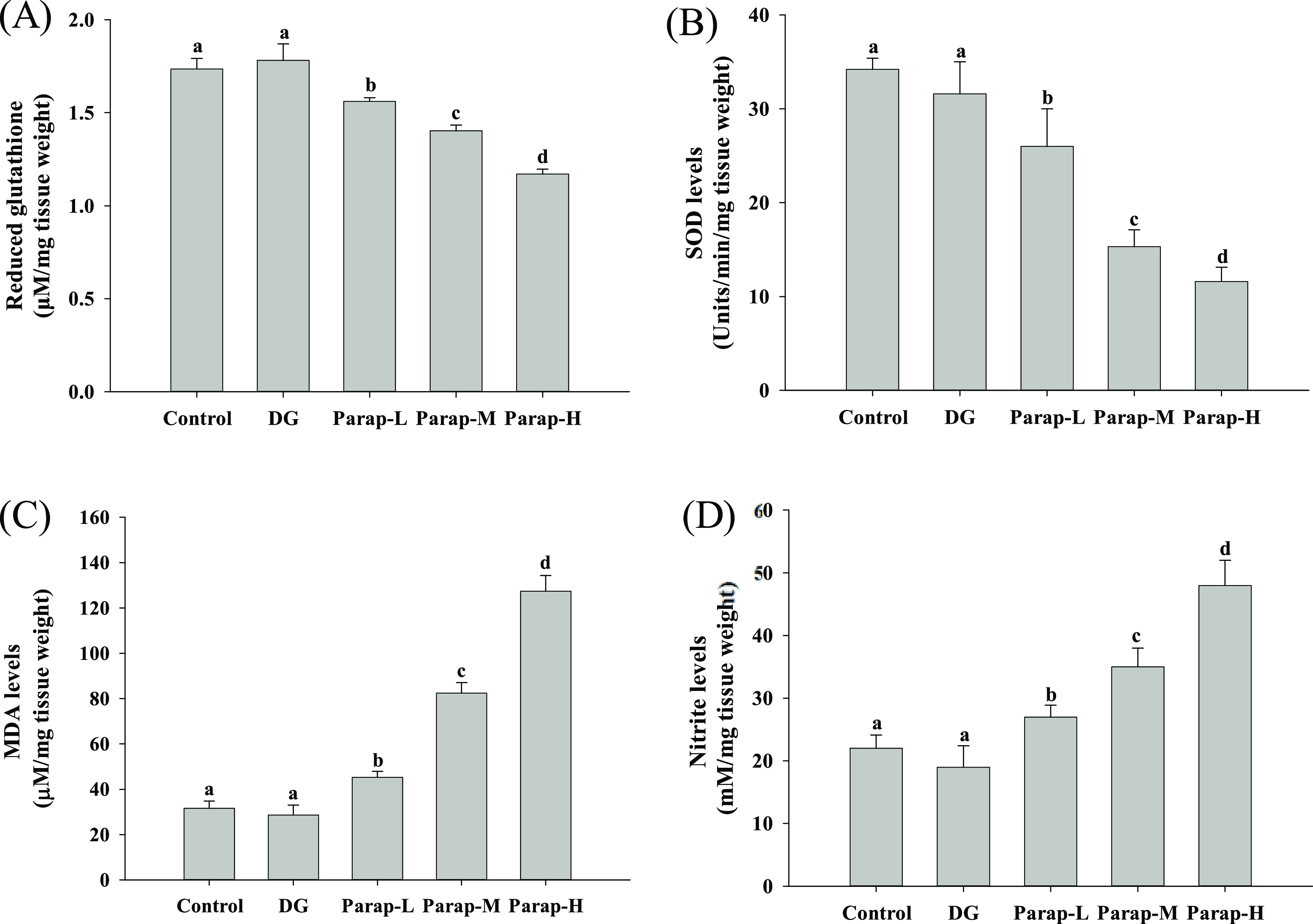
Effects of parapyruvate and DG on oxidative stress in brain tissues of the C57BL/6Narl mice. After being administered with low (Parap-L), medium (Parap-M), and high (Parap-H) dosages of parapyruvate and 120 mg/kg body weight/day of the DG, the levels of (A) GSH, (B) SOD, (C) MDA, and (D) nitrite of the randomly selected mice (*n* = 5 mice/group) were measured and are as shown. Values (means ± SD) not sharing an alphabetic letter are significantly different (*p* < 0.05).

The levels of GSH, MDA, and nitrite, and the SOD activity in the brain were analyzed to assess the oxidative stress. The results showed that parapyruvate significantly decreased the GSH level in a dose-dependent manner, as shown in the low, medium, and high dosages of the parapyruvate-administered groups and had 10.0, 19.2, and 32.6% lower GSH levels, respectively (Figure 6A). The SOD level lowered in 24.0, 55.3 and 66.1% for the low-, medium- and high-dose groups, respectively (Figure 6B). In addition, a significant dose-dependent increase in the MDA and nitrite levels was observed in mice treated with parapyruvate (Figure 6C,D). The MDA levels increased by 0.4, 1.6, and 3.0 times, and the nitrite levels increased by 0.2, 0.6, and 1.2 times for the Parap-L, Parap-M, and Parap-H groups, respectively. However, the mice administered with DG showed no significant effects on GSH, SOD, MDA, and nitrite. All of these results demonstrated that parapyruvate could induce oxidative stress in the brain.

### 2.6 Effects of Parapyruvate on the AChE Activity in the Brain

**Figure 7:**
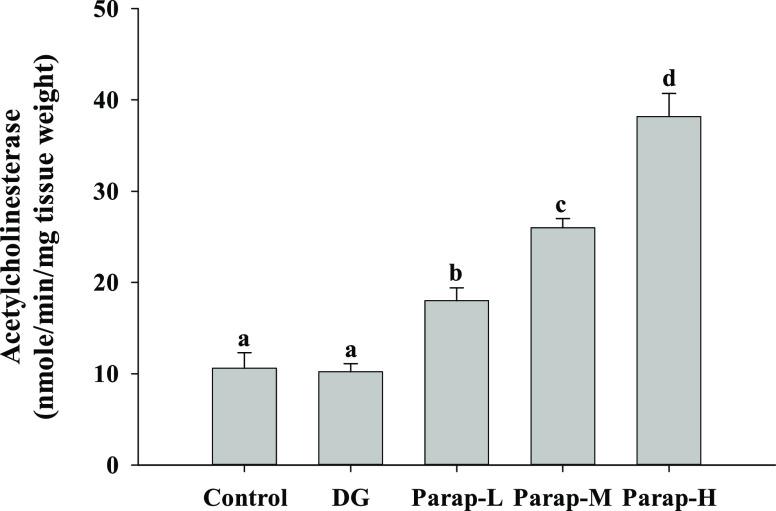
Effects of parapyruvate and DG on the acetylcholinesterase (AChE) activity in brain tissues of the C57BL/6Narl mice. After being administered with low (Parap-L), medium (Parap-M), and high (Parap-H) dosages of parapyruvate and 120 mg/kg body weight/day of DG, the AChE activity of the randomly selected mice (*n* = 5 mice/group) was measured and is as shown. Values (means ± SD) not sharing an alphabetic letter are significantly different (*p* < 0.05).

The results revealed that the AChE activity in the brain was significantly increased in the parapyruvate-treated group in a dose-dependent manner (Figure 7). However, the DG group showed no significant changes in the AChE activity in the brain compared with the control (Figure 7). The AChE activity in the brain increased by 0.7, 1.5, and 2.6 times in mice administered with low, medium, and high doses of parapyruvate, respectively.

### 2.7 Effects of Parapyruvate on the Expression Levels of Aβ42, Tau, p-Tau, and MBP in the Brain

**Figure 8:**
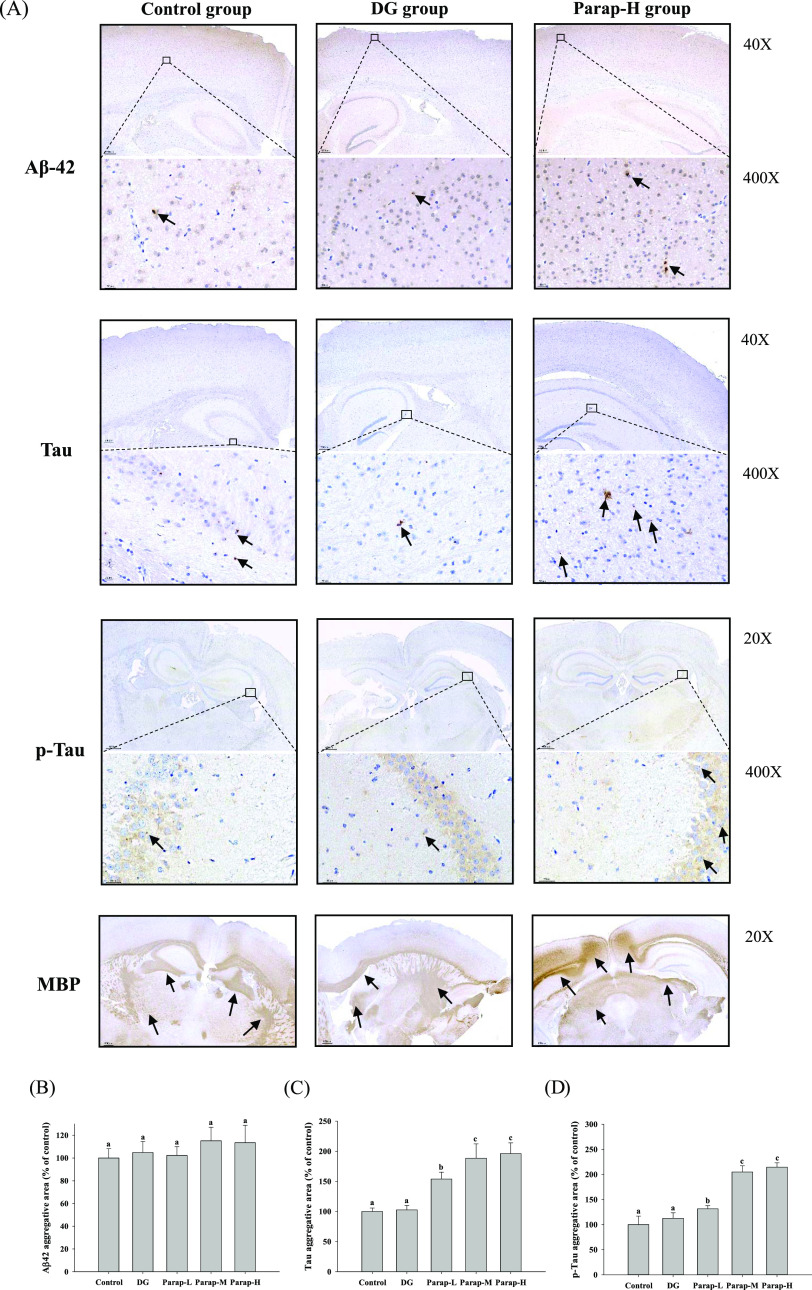
Effects of parapyruvate and DG on the expression levels of Aβ42, Tau, p-Tau, and myelin basic protein (MBP) in the cortex and hippocampus of the C57BL/6Narl mice. After being administered with low (Parap-L), medium (Parap-M), and high (Parap-H) dosages of parapyruvate and 120 mg/kg body weight/day of DG, the expression levels of these proteins were detected by immunohistochemical staining as described in the Methods section (*n* = 5 mice per group). The images shown are taken at 20×, 40×, 200×, or 400× magnification, with the aggregation of the (A) Aβ42, Tau and p-Tau, and MBP signals indicated by arrows. The (B) Aβ42, (C)Tau, and (D) p-Tau aggregative areas compared to the control group were determined and are as shown.

As shown in Figure 8A, the results showed that there were very small amounts of dot-like strong positive signals of aggregated Aβ42 scattered in the cortex and hippocampus in all of the groups, but there was no difference between the groups. For example, the aggregated Aβ42 signal in the cortex was quantified and showed having no significant difference in all of the groups (*p* > 0.05) (Figure 8B). As shown in Figure 8A, the aggregated Tau and p-Tau signals were strong and mainly seen in the cytoplasm of neurons or in the neuropil with a dot-like distribution. The signal quantity of Tau and p-Tau increased with the dosage of parapyruvate, but DG showed no significant effect on both signals (Figure 8C,D). In addition, MBP is normally expressed in nerve fibers, and it is expressed in large quantities in the white matter of the normal brain tissue. As shown in Figure 8A, the results showed that MBP was generally expressed in all of the brain section areas, but its expression was significantly stronger in the Para-H group than in the control and other groups.

## 3 Discussion

Previously, we have shown that parapyruvate is a reversibly competitive inhibitor of the KGDHC activity and can induce senescence in Hs68 cells by inhibiting the cellular KGDHC activity.^2,5^ In this study, we administered parapyruvate to the C57BL/6JNarl mice at dosages ranging from 5 to 500 mg/kg/day and observed a dose-dependent impairment of the learning and memory ability. Furthermore, we observed that parapyruvate both decreased the KGDHC activity and increased the oxidative stress in the brain in a dose-dependent manner, as evidenced by the decreased levels of GSH and the SOD activity and the increased levels of MDA and nitrite in the brain. In addition, parapyruvate increased the AChE activity in the brain in a dose-dependent manner and induced the aggregation of Tau and p-Tau in the cortex and hippocampus. At a dosage of 500 mg/kg/day, parapyruvate induced the MBP expression in the cortex and hippocampus in the mice brain. Even a low dosage of 5 mg/kg/day, which is 10 times lower than the equivalent dosage of 50 mg/kg/day, was found to significantly inhibit the KGDHC activity and induce the learning impairment, oxidative stress, Tau and p-Tau aggregation, and AChE activity. Taken together, these findings indicated that the long-term administration of the equivalent dosage of parapyruvate can induce neurodegeneration in the C57BL/6JNarl mice, supporting our hypothesis that parapyruvate could inhibit the activity of KGDHC so as to induce oxidative stress, ultimately leading to brain aging and neurodegeneration. Importantly, this article is the first to demonstrate the neurotoxicity of parapyruvate in an animal study.

The enzyme AChE primarily exists in the neuromuscular junction and cholinergic nervous system where it acts as a hydrolase to break down the neurotransmitter acetylcholine (ACh) into acetate and choline.^45^ Ach has been demonstrated to play an important role in the learning and memory ability.^46−48^ Overactivity of AChE causes a decrease in ACh concentration, leading to the degeneration of the cholinergic system and inducing cognition loss.^45^ AChE is also involved in the pathogenesis of neurodegenerative diseases by influencing the inflammatory response, apoptosis, oxidative stress, and aggregation of pathological proteins.^45^ Therefore, AChE inhibitors can be used to improve the lives of patients with AD.^49−51^ Additionally, it has been mentioned that the activity of AChE can serve as a biomarker of brain neurotoxicity, which increases through elevating oxidative stress in rats.^52^ In this study, the results demonstrated that parapyruvate could induce an increase in oxidative stress and AChE activity in the brain, supporting the notion that the increase in oxidative stress plays a crucial role in the parapyruvate-induced neuronal injury.

Various etiological mechanisms have been proposed for AD, including the decrease of the KGDHC activity, the deposition of Aβ, the aggregation of Tau protein, the increase of oxidative stress, and the reduction of ACh.^21,53^ In this study, our results suggest that the decrease in the KGDHC activity and the increase in the oxidative stress may share a common mechanism for the etiology of AD. As shown, we found that the KGDHC inhibitor parapyruvate significantly increased the level of oxidative stress. Moreover, it appears that the aggregation of Tau protein, the reduction of ACh, and the deposition of Aβ are also related to high oxidative stress. High oxidative stress has been shown to promote the deposition of Aβ42^54^ and lead to the hyperphosphorylation of Tau protein, which could result in the instability and dissociation of the microtubules in the brain and ultimately lead to the oligomerization of Tau protein.^54^ The oxidative stress has also been considered a key modulator in neurodegenerative diseases, including AD.^30^ In this study, the results showed that parapyruvate could induce the aggregation of Tau and p-Tau, and the increase of the AChE activity, supporting the crucial role of the oxidative stress in the etiology of AD. However, we did not observe significant aggregation of Aβ42 in the brain tissue of the C57BL/6JNarl mice induced by parapyruvate.

The neurotoxicity of DG is commonly used as a model for drug screening for the brain aging,^40,41,55−58^ or for AD.^59,60^ The continuous subcutaneous administration of DG in mice was also found to induce an increase in the AChE activity in the brain.^59^ However, in this study, we used 120 mg/kg/day of DG administered subcutaneously 5 times per week, and the results showed that DG only significantly decreased the KGDHC activity in the C57BL/6JNarl mice. We were unable to obtain all of the positive indicators reported in the literature. In fact, we found that the appropriate dosage of DG to induce aging in mice appeared to be different for different strains of mice.^55,56,60,61^ Previous studies have suggested that the appropriate dosage of DG to induce aging in the C57BL/6JNarl mice is 50–100 mg/kg/day administered subcutaneously for 8 weeks,^62^ but most of the studies used 120 mg/kg/day.^63,64^ However, one study showed that administering DG at a dosage of 125 mg/kg/day to the C57BL/6JNarl mice for 8 weeks caused significant handgrip loss but did not impair the learning and memory ability,^65^ indicating that the dosage used to induce aging by DG in the same strain of mice may remain inconclusive. In this study, we used 120 mg/kg/day of DG administered subcutaneously 5 times per week. It is possible that the frequency of DG administration used in this study was insufficient to obtain all of the positive indicators, as reported in the literature.

We were also interested in investigating whether the neurotoxicity of parapyruvate could be used as a model for drug screening for brain aging, including AD, in mice. In this study, we found that the neurotoxic effects of parapyruvate appear to be stronger than those of DG even though the dosages used and the routes of administration were different. While our used dosage of DG only significantly decreased the KGDHC activity, the effects of our used dosages of parapyruvate on physical behaviors and biochemical indicators, including the induction of oxidative stress, were more potent. The results demonstrated that the neurotoxic effects of parapyruvate could strongly induce brain aging and neurodegeneration. Therefore, we proposed a new model for brain aging by using parapyruvate administration for drug screening for the types of brain aging or neurodegeneration induced by the decrease of the KGDHC activity or the increase of oxidative stress. The advantage of the parapyruvate model for brain aging is that it can be administered orally, which is more convenient than the subcutaneous injection like DG. In addition, parapyruvate has recently become commercially available in the market. However, the administration of parapyruvate only induced one of the hallmarks of AD, i.e., the aggregation of Tau protein. The other hallmark of AD, the disposition of Aβ, was not found in this study. Therefore, further verification is necessary to determine whether the administration of parapyruvate is suitable as an animal model for AD. At least, we did not measure the disposition of other peptide lengths of Aβ in this study. Further studies are required to reveal whether parapyruvate could induce the disposition of Aβ.

Moreover, it was found that a high dosage of parapyruvate had the ability to induce the expression of MBP in the mouse brain. MBP is a major protein component of myelin in the human central nervous system^66^ and is an oligodendrocyte-specific protein necessary for the oligodendrocyte morphogenesis at the late stages of cell differentiation.^67^ In addition, MBP is a cellular marker of oligodendrocytes, an important protein for nerve myelination in the central nervous system,^43^ and interacts with lipids in the myelin membrane to maintain the proper structure of myelin.^68^ Massive degradation of MBP has been considered to be one of the major mechanisms in the pathogenesis of multiple sclerosis, which is an autoimmune neurodegenerative disease.^69^ However, it has been found that increase in the MBP expression in the rat’s brain during the acute injury period represents the proliferation of oligodendrocytes due to the compensatory proliferative response of oligodendrocytes to the oxidative stress.^43^ This study discovered that a high dosage of parapyruvate induced a greater amount of MBP expression in the brain tissue, suggesting that the high level of oxidative stress induced by parapyruvate may further induce the proliferation of oligodendrocytes.

Furthermore, Sayehmiri et al.^70^ found that succinyl phosphonate (SP), another KGDHC inhibitor and the phosphonate analogue of α-KG, could restore the reduced KGDHC activity and the cognitive decline induced by the early stage Aβ toxicity. Additionally, they observed that daily injection of a high dosage of SP cumulatively reached a toxic level that impaired the learning and memory ability in rats.^70^ In this study, since we did not expose the mice to Aβ, we did not observe any beneficial effects with the low-dosage administration of parapyruvate. However, the usage of a high dosage of SP supported our findings that KGDHC inhibition could impair the learning and memory ability in mice. In addition, the results demonstrated the neurotoxic effects of parapyruvate, indicating its capability to cross the BBB. However, the detailed mechanisms by which parapyruvate crosses the BBB remain to be further investigated. Furthermore, the significance and mechanism by which medium- and high-dosage parapyruvate can increase the body weight of mice also warrant further investigations.

However, can the results obtained in this study lead to the conclusion that the consumption of commercial calcium pyruvate may promote neurodegeneration and cognitive decline? It is important to note that our study administered pure parapyruvate to animals, while humans consume calcium pyruvate supplements that contain parapyruvate as an impurity. Previously, we found that the parapyruvate content is 1.4 ± 0.1 to 10.6 ± 0.2% in five brands of calcium pyruvate supplements.^2^ Excluding the weight of the excipients in the commercial DSCP, the estimated parapyruvate content in the commercial DSCP is approximately 10 to 20% of the calcium pyruvate content. As a result, humans receive a relatively small amount of parapyruvate in combination with a substantial amount of calcium pyruvate. It is essential to consider that pyruvate itself has known neuroprotective,^75,76^ antioxidant,^74^ and antiaging effects, which might counteract the potential negative effects of parapyruvate on the brain. Additionally, calcium ions have been shown to counteract the parapyruvate-induced senescence in Hs68 cells, as mentioned in our previous study.^2^ Thus, while this study demonstrates the neurotoxicity of pure parapyruvate, it cannot definitively conclude a safety concern in DSCP containing parapyruvate. Therefore, further studies will focus on the question of whether parapyruvate and calcium pyruvate interact differently when administered together in humans. We will administer an equivalent animal dosage of calcium pyruvate containing parapyruvate impurity to shed some light on the possible interactions and their impact on neurodegeneration in future studies.

In conclusion, this study demonstrated that the administration of parapyruvate significantly inhibits KGDHC activity, induces oxidative stress, increases AChE activity, promotes the aggregation of Tau and p-Tau, and impairs the learning and memory ability in the C57BL/6JNarl mice. All of these effects were observed at an equivalent dosage of 50 mg/kg/day of parapyruvate, indicating that there is a safety concern when taking calcium pyruvate dietary supplements containing parapyruvate. These results suggest that parapyruvate increases oxidative stress by inhibiting KGDHC activity and further induces brain aging and neurodegeneration in the C57BL/6JNarl mice. In addition, we propose that administering a dosage of parapyruvate higher than 50 mg/kg/day to the C57BL/6JNarl mice for more than 10 weeks would be suitable as an animal model for screening drugs for brain aging and neurodegeneration induced by decreased KGDHC activity or increased oxidative stress. However, further investigation is needed to determine whether the parapyruvate-induced brain aging mouse model is suitable for AD research.

## 4 Materials and Methods

### 4.1 Chemicals

Chemicals including d-galactose (DG), trichloroacetic acid, 2-thiobarbituric acid (TBA), malondialdehyde (MDA), Griess reagent, sodium nitrite, 5,5′-dithiobis(2-nitrobenzoic acid) (DTNB), reduced glutathione, Tris Base, ethylenediaminetetraacetic acid (EDTA), TritonX-100, pyrogallol, and acetylthiocholine iodide were all purchased from Sigma Chemical Company (St. Louis, MO). Pyruvic acid (>98%) was obtained from Alfa Aesar (Heysham, England). Acetone was purchased from Macron Fine Chemicals (Center Valley, PA). Ethanol was purchased from J.T.Baker Chemicals (Phillipsburg, NJ). NaCl, KCl, KOH, HCl, Na_2_HPO_4_, and KH_2_PO_4_ were obtained from Merck (Darmstadt, Germany). Parapyruvate was produced as referenced by the method from our previous study with a purity of ≥99% (5).

### 4.2 Calculation of the Equivalent Dosage of Parapyruvate for Mice

In our previous study, we determined the parapyruvate content in five brands of commercial DSCP. The brand with the highest parapyruvate content was brand 1, containing 10.6% parapyruvate.^2^ As each tablet of brand 1 weighed 2000 mg and the daily recommended dosage was one tablet, the maximum intake of parapyruvate in DSCP was estimated to be 212 mg/day. We used the conversion formula proposed by Reagan-Shaw et al.:^71^ the human equivalent dosage (mg/kg) = animal dosage (mg/kg) × (animal *K*_m_ value/human *K*_m_ value), where *K*_m_ is the body weight (kg) ÷ body surface area (m^2^); human *K*_m_ is approximately 37 and mouse *K*_m_ is 3. Thus, 12.3 times the human dosage is the mouse equivalent dosage. Then, the daily intake of 212 mg of parapyruvate for an adult body weight of 60 kg was converted into the dosage for mice, which was obtained as 44 mg/kg body weight/day. Therefore, the equivalent dosage for mice was set as 50 mg/kg/day in this study, and the low, medium, and high parapyruvate dosages were set as 5, 50, and 500 mg/kg body weight/day for mice.

### 4.3 Animal Experimental Design

Male C57BL/6JNarl mice (12 weeks old) weighing about 28 ± 2 g were used for experiments and were purchased from the National Laboratory Animal Center (Taipei, Taiwan). The mice were maintained on a 12 h light–dark cycle in a temperature-controlled room at 23–25 °C and provided with diet (LabDiet 5001 Rodent Diet, PMI Nutrition, St. Louis, MO) and water ad libitum throughout the study. All experimental procedures involving animals were conducted in accordance with the National Institutes of Health (NIH) guidelines, and the protocol was approved by the Institutional Animal Care and Use Committee (IACUC) of the Chung-Shan Medical University Experimental Animal Center (IACUC Approval No: 1666). DG was dissolved in 0.9% normal saline and administered by a single subcutaneous injection (SI) at a dosage of 120 mg/kg body weight/day,^63^ with a frequency of 5 times a week. Parapyruvate was administered with the LabDiet 5001 Rodent Diet. The mice were randomly divided into five groups (10 mice/group) as follows: Control, DG (120 mg/kg body weight/day; 5 days a week), parapyruvate-low dosage (Parap-L; 5 mg/kg body weight/day), parapyruvate-medium dosage (Parap-M; 50 mg/kg body weight/day), and parapyruvate-high dosage (Parap-H; 500 mg/kg body weight/day). The body weight of mice was recorded weekly, and the animal behavioral tests, comprising the MWM and active avoidance tests, were performed on days 57–59 and days 66–68, respectively. On day 69, the mice were sacrificed for subsequent analysis of brain tissues.

### 4.4 Morris Water Maze Task

The Morris water maze (MWM) task was performed based on our previous method^34^ with some modification and was used to assess the memory and learning ability, including the reference memory test and the probe test. A stainless-steel circular tank (diameter 160 cm, height 60 cm) was used as the equipment for the water maze in which a movable escape platform (diameter 10 cm, height 26 cm) was concealed inside the tank. The tank was filled to a height of 28 cm with approximately 23 ± 2 °C water and clouded with 4 L of milk. The circular tank was partitioned into four quadrants (R1, R2, R3, and R4), and a position with an equal distance from the center and edge in the middle of each quadrant was marked for the location of the platform. The water tank was located in a room with adjustable lighting; a camera was set on the ceiling above the center of the water tank, and we used the SINGA Real-Time Trace Mouse Ver. 1.17 System (Taipei, Taiwan) to record the behavioral performance of the mice.

#### 4.4.1 Reference Memory Test

The reference memory is an indicator of the learning ability of mice. A transparent platform was concealed in the R4 quadrant and submerged 1 cm below the water surface. During each trial, the mouse was placed in the maze at random positions in the R1–R4 quadrants and allowed to swim for 120 s. The latency of the mice to escape onto the hidden platform was recorded for each trial. If a mouse failed to reach the platform within 120 s, the training was terminated, and the escape latency was recorded as 120 s. In both situations, the mice were allowed to remain on the platform for 30 s before being returned to their home cage to rest for 60 min before the next trial. The training was conducted for three consecutive days, 4 times a day, from days 57 to 59, for a total of 12 trials. By means of the 12 trials of the reference memory test, the mice gradually recognized the location of the escape platform and exhibited a reduction in the escape latency.

#### 4.4.2 Probe Test

The probe test was conducted on day 59, 4 h after the completion of the reference memory tests, to ascertain whether the mice had retained the memory after the reference memory test. In this test, the hidden transparent platform was removed from the pool, and each mouse was placed into the pool from the R1 quadrant and allowed to swim for 60 s in the water maze. The test was carried out by recording the swimming route for each mouse in the pool and calculating the swimming distance and time to cross the R4 quadrant, where the hidden platform was located in the reference memory test but was removed in this test.

### 4.5 Active Avoidance Test

Active avoidance test was conducted to evaluate the learning and memory ability by using a Gemini Avoidance System (San Diego Instruments, San Diego, CA), with modification based on our previous study.^34^ The test measured the escape latency of successful avoidance, where a longer escape latency indicated poorer learning and memory ability. To begin with, each mouse was placed individually in the large compartment of the apparatus with the door closed and allowed to adapt for approximately 10 s. A 60 W light bulb was randomly switched on in the opposite compartment of the mouse and used as a conditioning stimulus (CS). The CS appeared 5 s prior to the commencement of the unconditioned stimulus (US), which was an electric shock (0.3 mA for 5 s) applied to the floor grid. If the animal avoided the US by running into the light compartment within 5 s after the onset of the CS, the microprocessor recorder unit of the shuttle box recorded an avoidance response. The escape latency was the time required for a mouse to move to the other compartment after the onset of the light stimulus. A “no response” was recorded if a mouse had no response after the onset of the light stimulus. Each mouse was given five trials daily for 3 days with an intertrial interval of 20 s. The recorder unit of the automated shuttle box continuously recorded this parameter during all experimental periods (15 trials). The results are expressed as the average time spent on the active avoidance responses for each daily test.

### 4.6 Determination of the KGDHC Activity

The KGDHC activity in the brain tissue homogenates was assayed using a commercial α-ketoglutarate dehydrogenase activity colorimetric assay kit (K678–100, Biovision). To begin with, 0.4 g of the brain tissue was rapidly homogenized with 400 μL of ice-cold KGDH assay buffer and kept on ice for 10 min. The homogenate was then centrifuged at 10,000*g* for 5 min, and the resulting supernatant was transferred to a fresh tube as the test sample. A 100 μL aliquot of the reaction mixture was prepared, which contained 50 μL of the sample, KDGH positive control or NADH standard, and 50 μL of the reaction mix, following the manufacturer’s protocol. After incubation for 10 min, the absorbance was measured immediately at 450 nm in a kinetic mode for 30 min at 37 °C. The KGDHC activity in the brain tissue was calculated using the following equation: sample α-ketoglutarate dehydrogenase activity = *B*/(Δ*T* × V) × D = mU/mL, where *B* is the NADH amount from the standard curve (nmol); Δ*T* is the reaction time (min); *V* is the sample volume added into the reaction well (mL); and *D* is the dilution factor.

### 4.7 Determination of the GSH Level

The GSH levels in the brain tissue homogenates were assayed according to the method described by Chavali et al.^72^ The brain tissue was homogenized in 5 mL of 0.2 M phosphate buffer (pH 7.6), and 0.1 mL of 25% trichloroacetic acid solution was added to the homogenate, which was then centrifuged for 10 min at 3900*g* at 25 °C. Next, 1 mL of supernatant and 1 mL of phosphate buffer (pH 7.6) were mixed together, followed by the addition of 1 mL of DTNB and vortexing. The samples were incubated for 5 min at room temperature, and the absorbance was measured at 412 nm against the blank DTNB absorbance. A standard curve was prepared by using different concentrations of the reduced glutathione standard.

### 4.8 Determination of the SOD Activity

The SOD activity was analyzed following the method described by Chavali et al.,^72^ with some modification. The whole brain tissue (0.4–0.42 g) was homogenized in 2 mL of chilled 50 mM Tris buffer (pH 8.2 with 2 mM EDTA) in three cycles of 30 s each at 13,000 rpm with a 30 s gap between each cycle. Then, 1 mL of 1% TritonX-100 was added to this homogenate, vortexed thoroughly, and incubated for 20 min at 4–8 °C before being centrifuged at 10,000 rpm at 4 °C for 30 min. The supernatant was collected and kept in a refrigerator for 45 min. Samples and pyrogallol absorbances were recorded every 60 s for 10 min at 420 nm. The reaction mixtures without the brain homogenate served as the control. The rate of increase in absorbance units (*A*) per minute for the control and the test samples was determined, and the following formula was used to estimate the percentage inhibition for the test samples:
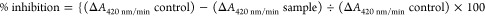
Units of the SOD activity were presented as the amount of enzyme required to inhibit the reduction of pyrogallol by 50%, and the activity was expressed in units per gram of the brain tissue.

### 4.9 Determination of the MDA Level

Lipid peroxidation was measured in the brain homogenates as previously described.^73^ Briefly, the brain tissues were weighed and homogenized with 10 times the volume of phosphate buffer (5 mM, pH 7.4). The homogenized brain tissue was centrifuged, and the resulting supernatant was mixed with 10% trichloroacetic acid and centrifuged at 8000*g*, 4 °C for 10 min. The supernatant was collected and incubated with 0.8% (w/v) TBA at 100 °C for 15 min. The absorbance was detected at 532 nm, with MDA (Sigma-Aldrich, St. Louis, MO) as a standard. The results were represented as μm of MDA/mg of tissue weight.

### 4.10 Determination of Nitrite Levels

The level of nitric oxide in brain tissues was determined by estimating the nitrite concentrations using the Griess reagent.^72^ The brain tissue was homogenized in 4 mL of ice-cold 0.2 M phosphate buffer (pH 7.6). 300 μL of the homogenized tissue sample was mixed with 100 μL of the Griess reagent and 2.6 mL of the phosphate buffer. A photometric reference was prepared by mixing 100 μL of the Griess reagent and 2.9 mL of 0.2 M phosphate buffer and incubating for 30 min at room temperature. The absorbance was recorded at 548 nm. The concentration of nitrite was calculated in micromolar per milligram of the brain tissue using a standard curve of sodium nitrite.

### 4.11 Determination of the AChE Activity

Acetylcholinesterase (AChE) activities in brain tissues were measured using acetylthiocholine iodide as a substrate with some modification.^52^ The reaction mixture contained 0.1 M phosphate buffer (pH 8) and 0.01 M DTNB. The hydrolysis rate of acetylthiocholine iodide was measured at 412 nm by monitoring the release of the thiol compound, which produced the color-forming compound DTNB upon reaction with DTNB. The reaction was initiated by adding 0.075 M acetylthiocholine iodide. Activities were expressed as nanomoles of the substrate hydrolyzed per minute per milligram of tissue weight.

### 4.12 Immunohistochemical Stain Analysis

The expression levels of Aβ42, Tau, p-Tau, and MBP in the cortex and hippocampus were determined by IHC staining, which was conducted by Lizhong Biotechnology INC (Kaohsiung, Taiwan). The wet tissues were trimmed and dehydrated using a serial alcohol solution, then embedded in paraffin wax, and cut into 3 μm thick sections. The tissue was dried at 60 °C for 1 h and then deparaffinized and hydrated through a series of alcohol solutions (100, 95, and 70% alcohol). Antigen retrieval was carried out using the boiling citrate buffer (pH 6.0) for 12 min, followed by the protein blocking with a blocking buffer (TA00C2, BioTnA, Kaohsiung, Taiwan) at room temperature for 60 min. The endogenous peroxidase activity was blocked using hydrogen peroxide for 20 min. The primary polyclonal antibody including Aβ42 (GeneTex, Hsinchu, Taiwan), Tau (BioSS, Massachusetts), p-Tau (Bioworld, Dublin), and MBP (Bioworld, Dublin) was incubated with 200 or 500× dilution at room temperature for 10 min. The protein expression level was determined by using the TnAlink mouse/rabbit polymer detection system (BioTnA, Kaohsiung, Taiwan) at room temperature for 30 min. The sections were stained with 3,3′-diaminobenzidine (DAB) for 5 min, then with hematoxylin for 3 min, and rinsed with running tap water for 10 min. Finally, the sections were dehydrated quickly through 95% ethyl alcohol and absolute ethyl alcohol, cleared in xylene, and mounted with a resinous mounting medium. All glass slides were observed and recorded under a Motic EasyScan (Motic Hong Kong Limited, Hong Kong, China) at 20, 40, 100, 200, and 400× magnifications to examine and document tissue lesions. Regarding the analysis of the staining results, three random fields of the cortex or hippocampus at 200× magnification were selected for capturing the images of Aβ42, Tau, and p-Tau. The captured images were subjected to digital image analysis using ImageJ. Color analysis in ImageJ was employed to calculate the percentage (%) of the IHC-positive signals in the captured images relative to the whole tissue areas. The obtained percentages for each group were compared to the control so as to calculate the % of the control (*n* = 5 mice per group). In addition, the MBP is a protein abundantly expressed in normal brain tissues, and its distribution varies significantly due to the differences in slice sampling and exhibited anatomical positions. Therefore, random field analysis using ImageJ was deemed unsuitable for MBP. Hence, only low-magnification images were presented.

### 4.13 Statistical Analysis

The animal body weight and behavioral data were analyzed by using the two-way repeated measures ANOVA, followed by the Tukey test.^77^ The biochemical data were analyzed by one-way ANOVA, followed by the least significant differences (LSD) test. All analyses relied on the use of SPSS v 17.0 (SPSS, Inc., Chicago, IL). *p* < 0.05 was considered statistically significant.

## Data Availability

Data can be provided upon request.

## Supporting Information Available

Swimming speed of mice in the Morris water maze test, and the effects of the parapyruvate and the DG on the body weight in the C57BL/6JNarl mice (PDF)

## Abbreviations

DSCP: dietary supplements of calcium pyruvate
KGDHC: α-ketoglutarate dehydrogenase complex
MWM: Morris water maze
AChE: acetylcholinesterase
MBP: myelin basic protein
α-KG: α-ketoglutarate
TCA: tricarboxylic acid cycle
AD: Alzheimer’s disease
PD: Parkinson’s disease
Aβ: amyloid β peptide
SOD: superoxide dismutase
GSH: glutathione
MDA: malondialdehyde
IHC: immunohistochemical
DG: d-galactose
Parap-L: parapyruvate-low
Parap-M: parapyruvate-medium
Parap-H: parapyruvate-high
ACh: acetylcholine
SP: succinyl phosphonate
TBA: trichloroacetic acid, 2-thiobarbituric acid
DTNB: 5,5′-dithiobis(2-nitrobenzoic acid)
EDTA: ethylenediaminetetraacetic acid
CS: conditioned stimulus
US: unconditioned stimulus
