# Supplementary material for: Parapyruvate Induces Neurodegeneration in C57BL/6JNarl Mice via Inhibition of the α-Ketoglutarate Dehydrogenase Complex
Source: ACS Omega. 2024 Jan 25;9(5):5919–31. doi: 10.1021/acsomega.3c09469 (PMC10851423; doi:10.1021/acsomega.3c09469)
Supplement: Supplementary file 1 — ao3c09469_si_001.pdf [file ao3c09469_si_001.pdf]

Parapyruvate induces the neurodegeneration in C57BL/6JNarl mice via inhibition of the α-ketoglutarate dehydrogenase complex

Inn Lee[a], Tuzz-Ying Song[b], Chien-Lin Chen[b], Jiann-Jou Yang[c], Nae-Cherng Yang[a,d,*]

[a]Chung Shan Medical University, Department of Nutrition, No. 110, Sec. 1, Jianguo N. Rd., Taichung 40201, Taiwan
[b]Da-Yeh University, Department of Medicinal Botanicals and Foods on Health Applications, No. 168, University Rd., Dacun, Changhua 51591, Taiwan
[c]Department of Biomedical Sciences, Chung Shan Medical University, No. 110, Sec. 1, Jianguo N. Rd., Taichung 40201, Taiwan
[d]Department of Nutrition, Chung Shan Medical University Hospital, No. 110, Sec. 1, Jianguo N. Rd., Taichung 40201, Taiwan

*To whom correspondence should be addressed. (Tel.: +886-4-3609-7673; Fax: +886-4-2324-8175; E-mail: naeman@csmu.edu.tw).

Supplementary Materials

Table S1. The swimming speeds of mice in the Morris Water Maze test

Group	Swimming speeds (mm/s)		
	Day 57	Day 58	Day 59
Control	203 ± 12.3[a]	189 ± 6.3[a]	206 ± 10.7[a]
DG	195 ± 8.9 [a]	199 ± 10.6[a]	187 ± 23.5[a]
Parap-L	210 ± 5.6 [a]	197 ± 8.7[a]	196 ± 14.8[a]
Parap-M	189 ± 13.7[a]	191 ± 10.8[a]	192 ± 15.2[a]
Parap-H	206 ± 5.8 [a]	192 ± 5.9[a]	203 ± 11.6[a]

Values (means ± SD) at the same column not sharing an alphabetic letter are significantly different ($p < 0.05$).

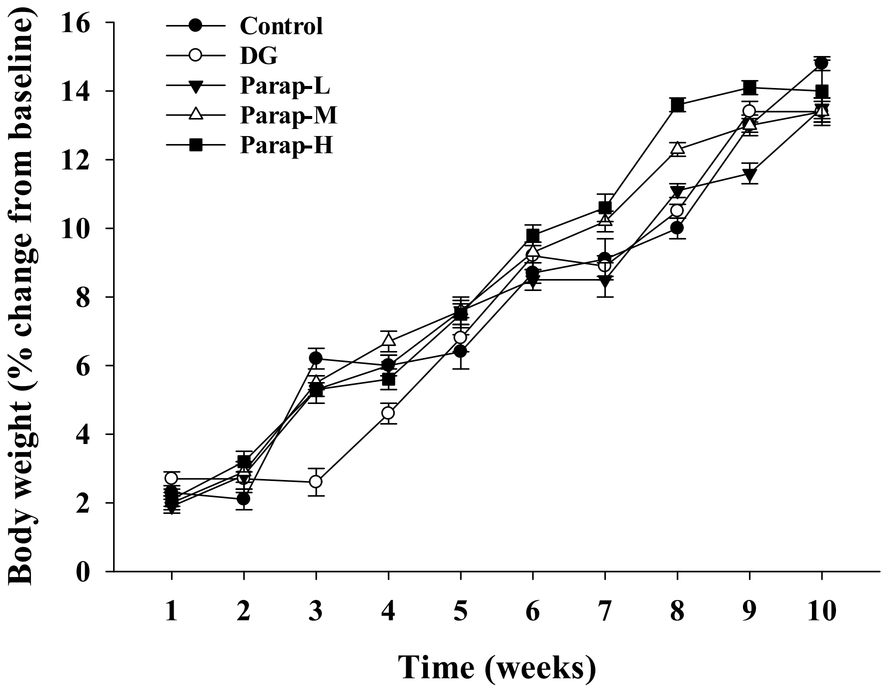

Figure S1. The effects of the parapyruvate and the DG on the body weight in the C57BL/6JNarl mice. Parapyruvate was administrated in the daily diet at the dosages of 5 (the low dose of parapyruvate; Parap-L), 50 (the medium dose of parapyruvate; Parap-M), and 500 mg/Kg body weight /day (the high dose of parapyruvate; Parap-H). The DG was dissolved in 0.9% normal saline and injected subcutaneously at a dosage of 120 mg/Kg body weight/day five days per week. The % changes from the baseline in the body weight during the administration for all the groups were shown. Data are expressed as means ± SD (n = 10 mice per group), and are analyzed by the two-way repeated measure ANOVA followed by the Tukey test.
